# Round-Efficient Secure Inference Based on Masked Secret Sharing for Quantized Neural Network

**DOI:** 10.3390/e25020389

**Published:** 2023-02-20

**Authors:** Weiming Wei, Chunming Tang, Yucheng Chen

**Affiliations:** 1 School of Mathematics and Information Science, Guangzhou University, Guangzhou 510006, China; 2 Guangzhou Center for Applied Mathematics, Guangzhou University, Guangzhou 510006, China; 3 School of Mathematics, Jiaying University, Meizhou 514015, China

**Keywords:** secure inference, quantized neural network, masked secret sharing

## Abstract

Existing secure multiparty computation protocol from secret sharing is usually under this assumption of the fast network, which limits the practicality of the scheme on the low bandwidth and high latency network. A proven method is to reduce the communication rounds of the protocol as much as possible or construct a constant-round protocol. In this work, we provide a series of constant-round secure protocols for quantized neural network (QNN) inference. This is given by masked secret sharing (MSS) in the three-party honest-majority setting. Our experiment shows that our protocol is practical and suitable for low-bandwidth and high-latency networks. To the best of our knowledge, this work is the first one where the QNN inference based on masked secret sharing is implemented.

## 1. Introduction

As an essential application of machine learning as a service (MLaaS) [1], neural network inference is widely used in image recognition [2,3], medical diagnosis [4], and so on. In the traditional MLaaS paradigm, the model owner provides a trained neural network, and a user, who holds some queries, calls an API of MLaaS to enjoy the inference service. However, with the increase in people’s privacy awareness and the perfection of laws and regulations [5], the traditional MLaaS paradigm is being challenged. On the one hand, the user is unwilling to reveal queries and inference results to the model owner. On the other hand, the trained model is intellectual property belonging to the model owner and cannot be revealed to the user. *Secure inference* utilizes cryptographic techniques to ensure that sensitive information is not revealed to each other.

In general, different cryptographic tools have different concerns. Fully homomorphic encryption (FHE) is communication-efficient but computation-expensive, which makes it unpractical [6]. As an important component of secure multiparty computation (MPC), secret sharing (SS) is computation-efficient but more communication rounds are required [7,8]. Existing works from secret sharing are usually under this assumption of the fast network, which has a high-bandwidth and low-latency network, for example, in the local area network (LAN) setting. However, all these works are inefficient in low-bandwidth and high-latency networks, even under the semi-honest model. A fast network is difficult to achieve in the real world, especially in the wide area network (WAN) setting. A proven method is to reduce communication rounds of the protocol as much as possible or construct protocols with constant rounds. In addition, these methods are also important for computationally intensive neural network inference.

Recently, QNN has gained much attention. The quantization technique reduces the overall model computational overhead by limiting the representation bit-width of data and parameters in the model at the expense of a certain level of model accuracy. More precisely, quantization converts the float-point arithmetic (FP32, 32-bit floating point, single precision) of neural networks into the fixed-point arithmetic (e.g., INT8, 8-bit fixed-point integer) since the latter is easy to deploy in resource-limited devices, such as laptops and mobile devices. We wonder the following: *could we achieve constant-round communication protocols based on secret sharing for QNN inference?* As we will show, the answer is yes with our proposed protocols.

### 1.1. Related Work on Secure Inference

Researchers in recent years have proposed several solutions for secure inference.

Gilad-Bachrach et al. proposed CryptoNets [6] mainly based on leveled homomorphic encryption, which allows a limited number of multiplication operations, and thus it is more efficient than the scheme based on FHE. However, the computation cost of CryptoNets is still high and unacceptable. Instead of relying on homomorphic encryption, some works introduce garbled circuit (GC) as the underlying cryptographic tool for secure inference. For example, DeepSecure [9] was the first work mainly based on GCs with free-XOR optimization, but it still has bad communication efficiency, even in low-latency networks. Some other works based on three-party replicated secret sharing (RSS) focus on obtaining high throughput of secure inference, such as ABY3 [7] and Falcon [8]. Most works use multiple protocols to achieve better performance. For example, Mohassel and Zhang presented SecureML [10], which utilizes additive secret sharing (ASS) for linear operations and GCs for piecewise-approximated activation.

There are some works related to QNN. According to the bitwidth of weight, a QNN can be binary neural network, ternary neural network, and other varieties. Riazi et al. presented XONN [11] for the binary neural network, where the values of weights and activations are restricted to the binary set {−1,+1}. The multiplication is replaced by XNOR operations, which can be computed by GCs. Ibarrondo et al. proposed Banners[12] for binary neural network inference based on three-party RSS. Zhu et al. proposed SecureBiNN [13] for binary neural network inference based on three-party RSS and three-party oblivious transfer (OT). Agrawal et al. proposed QUOTIENT [14] for ternary neural networks, where the weight is restricted to the ternary set {−1,0,+1}. Ternary multiplication can be done by using 1-out-of-2 OT. Dalskov et al. presented SecureQ8 [15] based on three-party RSS in the different threat models for INT8 quantization. Shen et al. proposed a practical secure two-party framework ABNN2 for arbitrary-bitwidth QNN inference [16]. A few works focus on the secure training of QNN, such as [17]. However, reduction in communication rounds is not considered in all these works.

### 1.2. Our Contributions

This work considers QNN inference with INT8 quantization in the honest majority setting. In detail, our contributions are described as follows:

- We provide a series of constant-round communication complexity secure protocols for QNN inference, including secure truncation, conversion, and clamping protocol. We achieve this by constructing protocols based on MSS.
- We give detailed proof of security in the semi-honest model. Concretely, our protocols are secure against one single corruption.
- The experiment shows that our protocols are practical and suitable for the high-latency network. Compared to the previous work for quantized inference, our protocols are 1.5 times faster in the WAN setting.

The remainder of this work is organized as follows. In Section 2, we define notations and primitives related to cryptographic tools, security model, neural networks, and quantization. In Section 3, we show the architecture for QNN secure inference. In Section 4, we give several building blocks of QNN inference and provide security analysis of our protocols. In Section 5, we provide our QNN structure. In Section 6, we implement our protocols and then report the experimental results. Finally, we conclude this work in Section 7.

## 2. Preliminaries

### 2.1. Basic Notations

At first, we define the notations used in this work in Table 1.

### 2.2. Threat Model and Security

In this work, we consider three non-colluding servers as the computing parties of MPC to execute secure inference tasks, where static, semi-honest adversary A corrupts only a single party during the protocol execution. The semi-honest adversary corrupts one of three parties and obtains its view (including its input, random tape, and received messages during the protocol execution), but follows the protocol specification exactly.

Our protocols rely on secure pseudo-random function (PRF), and thus, we can only provide security against a computationally bounded adversary; hence, all our protocols are computationally secure. Formally, we can define semi-honest security as follows:

**Definition** **1**. (Semi-honest Security [18]). Let Π be a three-party protocol in the real world, $F:{\left({\left\{0,1\right\}}^{*}\right)}^{3}\to {\left({\left\{0,1\right\}}^{*}\right)}^{3}$ be the ideal funcationality in the ideal world. We say Π securely computes F in presence of a single semi-honest adversary if for every corrupted party $P_{i}$ (i∈{0,1,2}) and every input $x\in {\left({\left\{0,1\right\}}^{*}\right)}^{3}$, there exists an efficient simulator Sim such that

(1) $$\left\{\mathrm{Sim}\left(x_{i},F_{i}\left(x\right)\right),F\left(x\right)\right\}\overset{c}{\equiv }\left\{{\mathrm{View}}_{i,\Pi }\left(x\right),{\mathrm{Output}}_{\Pi }\left(x\right)\right\},$$

*where $|x_{1}\left|=\right|x_{2}\left|=\right|x_{3}|$, ${\mathrm{View}}_{i,\Pi }\left(x\right)$ is the view of $P_{i}$, ${\mathrm{Output}}_{\Pi }\left(x\right)$ is the output of all parties, and $F_{i}\left(x\right)$ is the i-th output of F(x).*

In other words, a protocol Π is computationally secure in the semi-honest model, if and only if the view of the ideal world simulator and the view of the real world adversary is computationally indistinguishable.

### 2.3. Secret Sharing Semantics

Let *x* be the secret. Similar to [19], we use the following sharing in this work.

- 〈·〉-sharing: ASS among $P_{1}$ and $P_{2}$. The dealer samples random elements $x_{1},x_{2}\in_{R}Z_{2^{\ell }}$ as the shares of *x*, such that $x=x_{1}+x_{2}\;\mathrm{mod}\;2^{\ell }$ holds. The dealer distributes the shares to each party such that $P_{i}$ for i∈{1,2} holds $x_{i}$. For simplicity, we denote ${\langle x\rangle }_{i}$ as the additive shares of $P_{i}$, and $\langle x\rangle :=\left(x_{1},x_{2}\right)$.
- 〚·〛-sharing: MSS among all parties. The dealer samples random element $\lambda_{x}\in_{R}Z_{2^{\ell }}$, computes $m_{x}=x+\lambda_{x}\;\mathrm{mod}\;2^{\ell }$, and then shares $\lambda_{x}={\langle \lambda_{x}\rangle }_{1}+{\langle \lambda_{x}\rangle }_{2}$ among $P_{1}$ and $P_{2}$ by 〈·〉-sharing. The dealer distributes the shares to each party, such that $P_{0}$ holds $\left({\langle \lambda_{x}\rangle }_{1},{\langle \lambda_{x}\rangle }_{2}\right)$, $P_{1}$ holds $\left(m_{x},{\langle \lambda_{x}\rangle }_{1}\right)$, and $P_{2}$ holds $\left(m_{x},{\langle \lambda_{x}\rangle }_{2}\right)$. For simplicity, we denote $〚x{〛}_{i}$ as the masked shares of $P_{i}$, and $〚x〛:=(m_{x},{\langle \lambda_{x}\rangle }_{1},{\langle \lambda_{x}\rangle }_{2})$.

Table 2 summarizes the individual shares of the parties for the aforementioned secret sharing. It is easy to see that each party only misses one share to reconstruct the secret *x*.

The above steps can also be extended to $Z_{2}$ by replacing addition/subtraction with XOR and multiplication with AND. We use both $Z_{2^{\ell }}$ and $Z_{2}$ as the computation fields and refer to the shares as *arithmetic sharing* and *boolean sharing*, respectively. We denote the Boolean sharing with B in the superscript, which means the Boolean sharing of bit *b* is ${\langle b\rangle }^{B}$ and $〚b{〛}^{B}$ depending on the type of sharing semantics.

Note that both 〈·〉-sharing and 〚·〛-sharing satisfy the linearity property, which allows the parties to compute the linear combination of two shared values *non-interactively*. We only introduce the basic operations of MSS in this section. To reduce communication costs, $F_{\mathrm{Rand}}$ is used (cf. Appendix A).

Suppose that $P_{i}$ for i∈{0,1,2} holds the shares $〚x〛=\left(m_{x},{\langle \lambda_{x}\rangle }_{1},{\langle \lambda_{x}\rangle }_{2}\right),〚y〛=\left(m_{y},{\langle \lambda_{y}\rangle }_{1},{\langle \lambda_{y}\rangle }_{2}\right)$, and public constants c,d,e.

- For linear combination z=cx±dy±e, the parties locally compute its shares to be $〚z〛=\left(m_{z},{\langle \lambda_{z}\rangle }_{1},{\langle \lambda_{z}\rangle }_{2}\right)=\left(c\cdot m_{x}\pm d\cdot m_{y}\pm e,c\cdot {\langle \lambda_{x}\rangle }_{1}\pm d\cdot {\langle \lambda_{y}\rangle }_{1},c\cdot {\langle \lambda_{x}\rangle }_{2}\pm d\cdot {\langle \lambda_{y}\rangle }_{2}\right)$.
- For multiplication z=xy, we denote as functionality $F_{\mathrm{Mul}}$, then $\Pi_{\mathrm{Mul}}$ can be achieved as follows [19]:
  1. $P_{0}$ and $P_{1}$ locally sample random ${\langle \lambda_{z}\rangle }_{1}$ and ${\langle \gamma_{xy}\rangle }_{1}$ by using $F_{\mathrm{Rand}}$;
  2. $P_{0}$ and $P_{2}$ locally sample random ${\langle \lambda_{z}\rangle }_{2}$ by using $F_{\mathrm{Rand}}$;
  3. $P_{0}$ locally computes $\gamma_{xy}=\lambda_{x}\lambda_{y}$ and sends ${\langle \gamma_{xy}\rangle }_{2}=\gamma_{xy}-{\langle \gamma_{xy}\rangle }_{1}$ to $P_{2}$;
  4. $P_{i}$ for i∈{1,2} locally computes ${\langle m_{z}\rangle }_{i}=\left(i-1\right)m_{x}m_{y}-m_{x}{\langle \lambda_{y}\rangle }_{i}-m_{y}{\langle \lambda_{x}\rangle }_{i}+{\langle \lambda_{z}\rangle }_{i}+{\langle \gamma_{xy}\rangle }_{i}$;
  5. $P_{i}$ for i∈{1,2} sends ${\langle m_{z}\rangle }_{i}$ to $P_{3-i}$, who locally computes $m_{z}={\langle m_{z}\rangle }_{1}+{\langle m_{z}\rangle }_{2}$.

It is easy to see that the multiplication requires communication of at most 3ℓ bits and 2 rounds. Note that steps 1–3 are independent of the secret *x* and *y*, which can be improved by using the offline–online paradigm (see Section 3). In this way, the multiplication only requires 2ℓ bits and 2 rounds in the online phase.

The aforementioned scalar operation can be extended to tensor A or vector α by sharing the elements of A or α element-wise. We omit the detail here.

### 2.4. Neural Network

A neural network usually includes many linear and non-linear layers, all stacked on top of each other such that the output of the previous layer is the input of the next layer. We summarize the linear layers and non-linear layers as follows.

The linear layers usually include fully connected layer and convolution layer. Both can be computed by matrix multiplications and additions:

- The fully connected layer can be formulated as y=Wx+b, where y is the output of the fully connected layer, x is the input vector, W is the weight matrix and b is the bias vector.
- The convolution layer can be converted into computing the dot product of the matrix and vector, and then one addition as shown in [20]; thus, it can be formulated as Y=WX+B.

The non-linear layers introduce nonlinearity into neural networks and allow bound inputs to a fixed range, for example, evaluating the activation function. In this work, we only consider the rectified linear unit (ReLU) activation, which is defined as ReLU(x)=max(x,0).

### 2.5. Quantization

Although there are many different quantization methods [21], we only consider the linear quantization method proposed by Jacob et al. [22] in this work. This is because the linear quantization method only involves linear operations, which benefits constructing an SS-based MPC protocol.

For 8-bit quantization, 32-bit float-point α∈R is quantized as an 8-bit integer $a\in {\left[0,2^{8}\right)}_{Z}$. The relationship between α and *a* is a dequantized function $D_{S,Z}$:

(2) $$\alpha =D_{S,Z}\left(a\right)=S\cdot \left(a-Z\right),$$

where $S\in R^{+}$ is called *scale*, and $Z\in {\left[0,2^{8}\right)}_{Z}$ is called *zero-point*. As pointed out by Jacob et al. [22], both *S* and *Z* are determined at the training phase of the neural network; thus, (S,Z) is a constant parameter in the inference phase. We use a single set of quantization parameters for each activation array and weights array in the same neural network layer.

**Figure 1:**
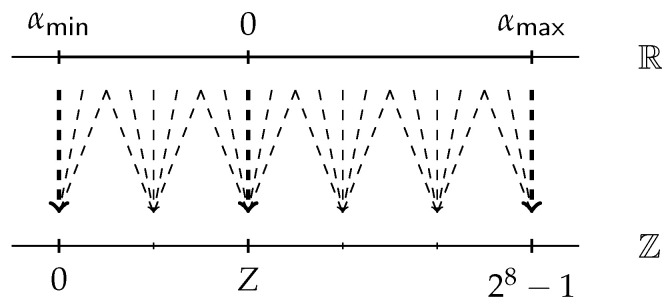
The visualization of quantized function [15], where $\alpha_{\min}=-S\cdot Z$, $\alpha_{\max}=S\cdot \left(2^{8}-1-Z\right)$.

In order to convert FP32 to INT8, we define quantized function $Q_{S,Z}$ to be the inverse of $D_{S,Z}$, then we have the following:

(3) $$a\approx Q_{S,Z}\left(\alpha \right)=\left\lfloor \frac{\alpha }{S}\right\rceil +Z,$$

where ⌊·⌉ is a rounding operation. Note that multiple numbers may map to the same integer due to the rounding operation; see Figure 1 (cf. [15]).

As an important part of QNN, when we compute the convolution of two quantized tensors, we have to compute the clamping function Clamp(x;a,b)=min(max(x,a),b) to bind the quantized result to ${\left[0,2^{8}\right)}_{Z}$, i.e., $\mathrm{Clamp}(x;0,2^{8}-1)$ should be computed. We refer the reader to [15,22] for more details.

## 3. The Architecture for Secure Inference

**Figure 2:**
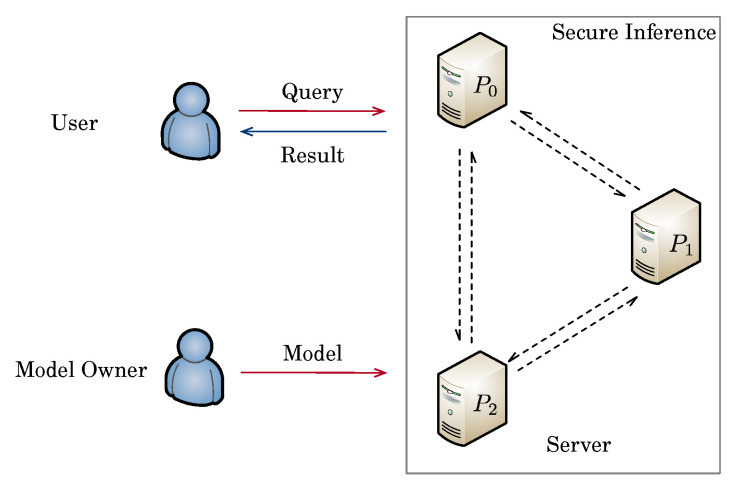
System architecture of secure inference.

Our secure inference system is built on outsourced computation architecture and is given in Figure 2. The system has three different roles, which we describe as follows:

- **Server**: There are three non-colluding servers in our system, denoted as $P_{1},P_{2},P_{3}$. Three servers can be from different companies in the real world, such as Amazon, Alibaba, and Google; any collusion will damage their reputations. Similar to prior works, we assume that all servers know the layer types, the sizes of each layer, and the number of layers. All servers perform a series of secure protocols proposed in Section 4 to execute inference tasks for users’ shared queries in a secure way.
- **User**: The user holds some queries as input and wants to enjoy a secure inference service without revealing both queries and inference results to others. To do so, the user uses Equation (3) to convert the query to the 8-bit integer firstly, then uses 〚·〛-sharing to split quantized queries to its masked shares before uploading to three servers, and receive the shares of inference results from three servers in the end. Note that only the user can reconstruct the final results; the privacy of both queries and inference results are protected during the secure inference.
- **Model Owner**: The model owner holds a trained QNN model, which includes all quantized weights of different layers along with the quantization parameters. As an important intellectual property belonging to the model owner, the privacy of the QNN model should be protected. To do so, the model owner uses 〚·〛-sharing to split quantized weights to its masked shares before deploying to three servers. Once the deployment is done, the model owner can go offline until the model owner wants to update the model.

Similar to prior works of secure inference [8,20], we do not consider black-box attacks toward neural networks, such as model extraction attacks, model inversion attacks, and membership inference attacks, since these attacks are independent of the cryptographic techniques used to make the inference process secure [23].

As pointed out by Dalskov et al. [15], we might not enjoy the benefits of the size reduction when considering secure inference. Although data and network weights can be stored by 8-bit integer, the arithmetic operation must be computed modulo $2^{\ell }$. This work only focuses on reducing communication rounds and computation costs among three servers.

We use the offline–online paradigm to construct our secure protocols. This paradigm makes it possible to split the protocol into the offline phase and online phase, where the offline phase is independent of the input of the parties and the online phase depends on the specific input. We argue that the user occasionally raises inference requests; the servers will have enough time to process the offline phase to speed up the execution of upcoming inference requests [23].

## 4. Protocols Construction

According to Section 3, the model owner provides the weights of the layer and the quantization parameters to three servers, which allows us not to consider the impact of quantization. To construct an efficient, secure inference scheme in the WAN setting, we need to create a series of building blocks with constant rounds communication for the three servers, which is the goal of this section. Our main contribution here is to present a secure truncation, conversion, and clamping protocol for secure inference of three servers. The other protocols follow the previous work [19], but we still give details for integrity.

### 4.1. Secure Input Sharing Protocol

Let $P_{i}$ be the secret owner holding *x*. We define functionality $F_{\mathrm{Share}}$, which allows the parties to generate 〚x〛. To achieve $F_{\mathrm{Share}}$, we follow [19] and show it in Protocol 1, which requires the communication of at most 2ℓ bits and 1 round in the online phase.


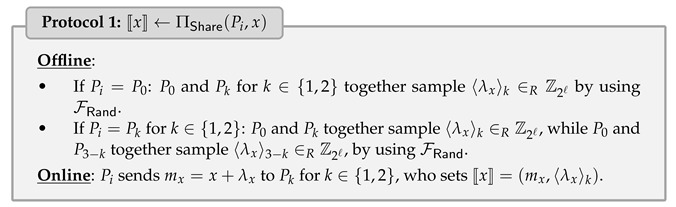


Observe that if both $P_{i}$ and $P_{j}$ hold the secret *x*, then $〚x〛:=(m_{x},{\langle \lambda_{x}\rangle }_{1},{\langle \lambda_{x}\rangle }_{2})$ can be generated without any communication by setting some shares to 0 instead of using $F_{\mathrm{Rand}}$, which is inspired by [24]. For simplicity, we still use the same notation to denote this case, i.e., $〚x〛\leftarrow F_{\mathrm{Share}}\left(P_{i},P_{j},x\right)$. To achieve $F_{\mathrm{Share}}$, $\Pi_{\mathrm{Share}}$ can be done as follows:

- $\left(P_{i},P_{j}\right)=\left(P_{0},P_{k}\right)$ for k∈{1,2}: The parties locally set $m_{x}={\langle \lambda_{x}\rangle }_{3-k}=0,{\langle \lambda_{x}\rangle }_{k}=-x$.
- $\left(P_{i},P_{j}\right)=\left(P_{1},P_{2}\right)$: The parties locally set $m_{x}=x,{\langle \lambda_{x}\rangle }_{1}={\langle \lambda_{x}\rangle }_{2}=0$.

### 4.2. Secure Truncation Protocol

Recall that when the parties execute secure multiplication protocol in the fixed-point value, we have to deal with the double-precision result. More precisely, when all shared values are represented as *ℓ*-bit fixed-point values with *d*-bit precision, then multiplying two fixed-point numbers, the result will be 2d-bit precision and must be truncated by *d* bits to keep right fixed-point representation. ABY3 [7] proposed the faithful truncation, which only works on RSS. Although [19] has the same semantics as us, they do not provide a secure truncation protocol in their work. In this work, we extend the faithful truncation to MSS as one of our contributions.

We define secure truncation functionality $〚x〛\leftarrow F_{\mathrm{Trunc}}\left(〚x^{\prime }〛,d\right)$, where $x^{\prime }$ has 2d-bit precision, and $x=x^{\prime }/2^{d}$. Suppose that the parties hold $〚x^{\prime }〛$ and random shared truncated pair $\left(r,r^{d}\right)$, where *r* is a random value, and $r^{d}$ denotes the value of the *r* truncated *d*-bit, i.e., $r^{d}=r/2^{d}$. The online phase of truncation can be performed by the parties to mask, reveal, truncate $\left(x^{\prime }-r\right)$ in the clear, use $r^{d}$ to unmask, and obtain the truncated result *x*, i.e., $x=\left(x^{\prime }-r\right)/2^{d}+r^{d}$.

The challenge here is to generate random shared truncated pair $\left(\langle r\rangle ,〚r^{d}〛\right)$ among the parties. To do so, we utilize the fact that if $r_{d}$ denotes the last *d* bits of *r*, then we have $r=2^{d}\cdot r^{d}+r_{d}$. Instead of sampling *r* by $P_{0}$ directly, $P_{0}$ and $P_{j}$ for j∈{1,2} together sample random ${\langle r\rangle }_{j}$ by using $F_{\mathrm{Rand}}$ such that $r={\langle r\rangle }_{1}+{\langle r\rangle }_{2}$ can be locally computed by $P_{0}$. In this way, $P_{0}$ can compute $r^{d}$ directly, and then share to $P_{1}$ and $P_{2}$ by invoking $F_{\mathrm{Share}}$. During the online phase, $P_{1}$ and $P_{2}$ reconstruct $y=x^{\prime }-r$ and truncate to obtain $y^{d}$, which follows by using $〚r^{d}〛$ to unmask the result. The protocol is described in Protocol 2, which requires the communication of at most 2ℓ bits and 1 round in the online phase.


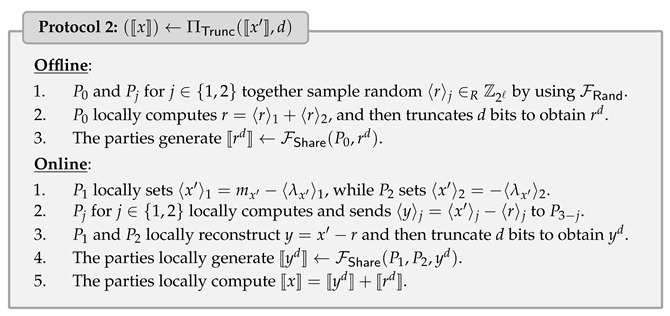


### 4.3. Secure Conversion Protocol

We define $F_{\mathrm{Bit}}2A$ to convert the Boolean shares of a single bit $〚b{〛}^{B}$ to its arithmetic shares 〚b〛. To do so, we utilize the fact that if *a* and *b* are two bits, then a⊕b=a+b−2ab.

Let $b^{A}$ be the value of bit *b* over $Z_{2^{\ell }}$, then according to the fact and masked sharing semantics, we have $b^{A}={\left(m_{b}⊕\lambda_{b}\right)}^{A}=m_{b}^{A}+\lambda_{b}^{A}-2m_{b}^{A}\lambda_{b}^{A}$, where $\lambda_{b}^{A}={\langle \lambda_{b}^{A}\rangle }_{1}⊕{\langle \lambda_{b}^{A}\rangle }_{2}={\langle \lambda_{b}^{A}\rangle }_{1}+{\langle \lambda_{b}^{A}\rangle }_{2}-2{\langle \lambda_{b}^{A}\rangle }_{1}\cdot {\langle \lambda_{b}^{A}\rangle }_{2}$. In other words, $\Pi_{\mathrm{Bit}}2A$ can be computed by invoking secure input sharing protocol and secure multiplication protocol of masked secret sharing. Note that $P_{0}$ holds both ${\langle \lambda_{b}^{A}\rangle }_{1}$ and ${\langle \lambda_{b}^{A}\rangle }_{2}$, and thus $u={\langle \lambda_{b}^{A}\rangle }_{1}\cdot {\langle \lambda_{b}^{A}\rangle }_{2}$ can be locally computed by $P_{0}$ without using Beaver triples.

To achieve $\Pi_{\mathrm{Bit}}2A$, we describe the construction in Protocol 3, which requires the communication of at most 2ℓ bits and 1 round in the online phase.


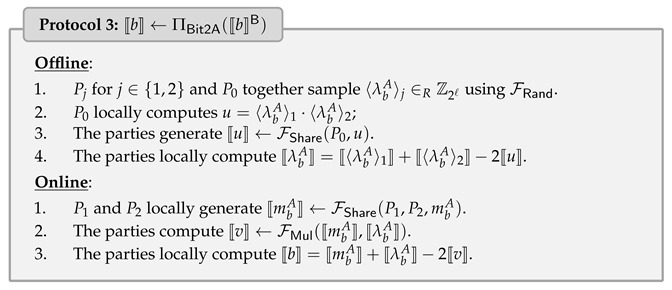


### 4.4. Secure Comparison Protocol

Comparison is an important building block of the neural network for evaluating ReLU activation, argmax function, and pooling layer. Fortunately, we can easily compare the quantized values if quantized *a* and *b* have the same quantization parameter (S,Z). This is because if α=S(a−Z) and β=S(b−Z), then α≤β holds if and only if a≤b holds. Therefore, the key step is to compute the most significant bit (MSB) of (a−b), i.e., a≤b if and only if MSB(a−b)=1. Letting x=a−b, we define secure comparison functionality $F_{\mathrm{MSB}}$ by giving shared value 〚x〛 and extract the Boolean shared bit $〚c{〛}^{B}$ such that $c=\left(a\leq_{?}b\right)=\mathrm{MSB}\left(x\right)$.

The secure comparison protocol of ABY3 [7] needs logℓ rounds in the online phase. To construct a constant-round comparison protocol, we implement $\Pi_{\mathrm{MSB}}$ with the three-party GC proposed by [19].

Let $GC(u_{1},u_{2},u_{3})$ be a GC with inputs $u_{1},u_{2}\in Z_{2^{\ell }},u_{3}\in \left\{0,1\right\}$, and output a masked bit $y=\mathrm{MSB}\left(u_{1}-u_{2}\right)⊕u_{3}$. We treat $P_{0}$ and $P_{1}$ as the common garbler and $P_{2}$ as the evaluator. The circuits are generated by $P_{0}$ and $P_{1}$ with correlated randomness by using $F_{\mathrm{Rand}}$. Namely, both garblers hold the knowledge of GCs, including the keys and the decoding table in clear. In our situation, the parties hold $〚x〛:=(m_{x},{\langle \lambda_{x}\rangle }_{1},{\langle \lambda_{x}\rangle }_{2})$; thus, we can define $u_{1}=m_{x}-{\langle \lambda_{x}\rangle }_{1}$ as the input of $P_{1}$, $u_{2}={\langle \lambda_{x}\rangle }_{2}$ as the input of $P_{2}$, and $u_{3}$ as a random bit sampled by $P_{0}$ and $P_{1}$ using $F_{\mathrm{Rand}}$.

Note that $P_{0}$ also knows $u_{2}$ and the corresponding key; hence, $P_{0}$ sends the key of $u_{2}$ to $P_{2}$ directly without using OT. $P_{2}$ evaluates the circuit to obtain *y*, then shares it with $\Pi_{\mathrm{Share}}$, which only requires communication of at most 2 bits. Finally, the parties remove masked bit $〚u_{3}{〛}^{B}$ to obtain masked share $〚c{〛}^{B}=〚\mathrm{MSB}(x){〛}^{B}$.

As pointed out by [25], the underlying circuit can be instantiated using the depth-optimized parallel prefix adder (PPA) of ABY3 [7]. GC can be further optimized by state-of-the-art techniques, such as free-XOR [26] and half gates [27]. We describe the details in the following Protocol 4, which requires the communication of at most κℓ+2 bits and 2 rounds in the online phase.


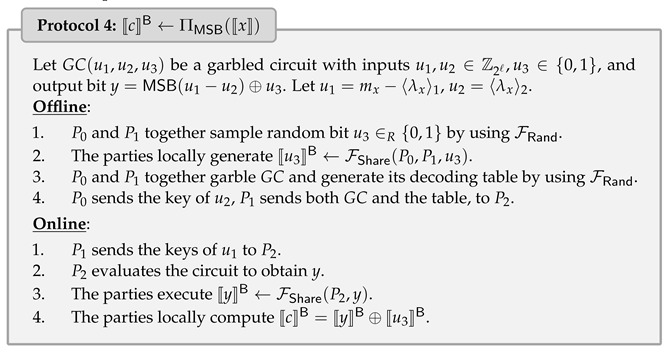


### 4.5. Secure Clamping Protocol

As pointed out by Section 2.5, when we compute the convolution of two quantized tensors, since rounding error exists, we may obtain the result $c\notin {\left[0,2^{8}\right)}_{Z}$, and hence a clamping operation $c\leftarrow \mathrm{Clamp}(c;0,2^{8}-1)$ should be computed [15].

Let $u=(x\leq_{?}a)$, then according to y=max(x,a), one has

(4) $$y=\left\{\begin{array}{cc} x, & \mathrm{if}\;u=0,\\ a, & \mathrm{if}\;u=1, \end{array}\right.$$

which is equivalent to the following Equation (5):

(5) y=(1−u)x+ua=x+u(a−x).

Similarly, let $v=(y\leq_{?}b)$, then according to z=min(y,b), one has the following Equation (6):

(6) z=(1−v)b+vy.

From Equations (5) and (6), one has

(7) $$\begin{array}{cc} z & =(1-v)b+v(x+u(a-x))\\ \; & =b+v(x-b)+uv(a-x), \end{array}$$

and thus the key point of the secure clamping protocol here is how to securely implement Equation (7).

Let e=x−b and f=a−x. Note that when we implement Equation (7) with masked secret sharing, both the shares of *u* and *v* are Boolean shares over $Z_{2}$, while both the shares of *e* and *f* are arithmetic shares over $Z_{2^{\ell }}$. In other words, we cannot invoke the secure multiplication protocol directly. This can be done by converting Boolean shares to arithmetic shares using secure conversion protocol and invoking secure multiplication protocol.

For simplicity, we formalize the above steps to be the bit injection functionality $〚c〛\leftarrow F_{\mathrm{BitInj}}\left(〚b{〛}^{B},〚x〛\right)$: given the Boolean shares of a bit *b* and the arithmetic shares of *x*, secure bit injection functionality allows the parties to compute c=bx. We provide $\Pi_{\mathrm{BitInj}}$ in Protocol 5, which requires the communication of at most 4ℓ bits and 2 rounds.

Now, we can give our secure clamping protocol in the following Protocol 6. Steps 5–6 can be computed in parallel within 2 rounds. Therefore, Protocol 6 requires the communication of at most 2κℓ+12ℓ+4 bits and 8 rounds in the online phase.


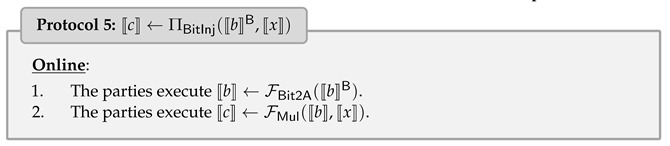



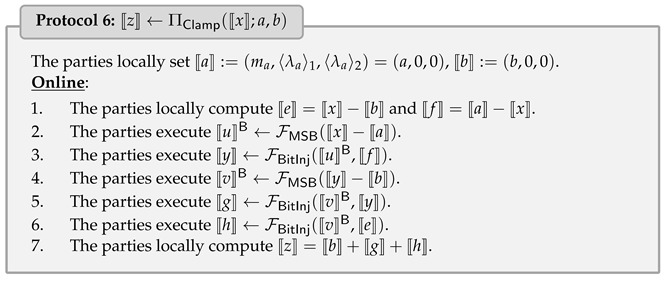


### 4.6. Theoretical Complexity

The total communication and round complexity of our protocols are provided in Table 3. It is easy to see that all our protocols have constant-round communication in the online phase.

### 4.7. Security Analyses

This section gives proof sketches of our protocols in the real–ideal paradigm. We present the steps of the simulator Sim for A in the stand-alone model with security under sequential composition [28]. The proof works in the $F_{\mathrm{Rand}}$-hybrid model.

**Theorem 1**. *$\Pi_{\mathrm{Share}}$ securely realizes the functionality $F_{\mathrm{Share}}$ in the $F_{\mathrm{Rand}}$-hybrid model and against a semi-honest adversary A, who only corrupts one single party.*

**Proof.**  Given the ideal $F_{\mathrm{Rand}}$, the output of PRFs is a pseudo-random value, which can be simulated by Sim uniformly samples random value. Note that $P_{i}$ sends $m_{x}$ to $P_{j}$, $m_{x}$ is masked by random ${\langle \lambda_{x}\rangle }_{i}$, which is unknown to $P_{j}$; hence, corrupted $P_{j}$ cannot learn any information of *x*. In short, the view of A in real execution is computationally indistinguishable from the view of Sim in ideal execution.    □

**Theorem 2**. *$\Pi_{\mathrm{Trunc}}$ securely realizes the functionality $F_{\mathrm{Trunc}}$ in the $\left(F_{\mathrm{Rand}},F_{\mathrm{Share}},F_{\mathrm{Mul}}\right)$-hybrid model and against a semi-honest adversary A, who only corrupts one single party.*

**Proof.**  Given ideal $F_{\mathrm{Rand}}$, $F_{\mathrm{Share}}$ and $F_{\mathrm{Mul}}$, the correlated randomness can be simulated by invoking $F_{\mathrm{Rand}}$. Then, Sim invokes $F_{\mathrm{Share}}$ to simulate step 4 in the offline phase. Finally, Sim invokes $F_{\mathrm{Mul}}$ to simulate step 2 in the online phase. Note that all functionality of the output is the random shares over $Z_{2^{\ell }}$, and hence the view of A in the real execution is computationally indistinguishable from the view of Sim in the ideal execution.    □

**Theorem 3**. *$\Pi_{\mathrm{Bit}}2A$ securely realizes the functionality $F_{\mathrm{Bit}}2A$ in the $\left(F_{\mathrm{Rand}},F_{\mathrm{Share}},F_{\mathrm{Mul}}\right)$-hybrid model and against a semi-honest adversary A, who only corrupts one single party.*

**Proof.**  The security of $\Pi_{\mathrm{Bit}}2A$ can be reduced to the security of $\Pi_{\mathrm{Share}}$ and $\Pi_{\mathrm{Mul}}$, which was proven to be secure in Theorem 1 and [19], respectively. Since we make only black-box access to $F_{\mathrm{Share}}$ and $F_{\mathrm{Mul}}$, according to the sequential composition, the Bit2A protocol is secure in the semi-honest model.    □

**Theorem 4**. *$\Pi_{\mathrm{MSB}}$ securely realizes the functionality $F_{\mathrm{MSB}}$ in the $\left(F_{\mathrm{Rand}},F_{\mathrm{Share}}\right)$-hybrid model and against a semi-honest adversary A, who only corrupts one single party.*

**Proof.**  Given the ideal functionality $F_{\mathrm{Rand}},F_{\mathrm{Share}}$, the security of $\Pi_{\mathrm{MSB}}$ is trivial for $P_{0}$ and $P_{1}$. This is because both $u_{1}$ and $u_{2}$ are unknown to $P_{0}$ and $P_{1}$ at the same time. Because the parties are non-colluding, we have that *y* is oblivious to the corrupted party, even if both garblers have the circuit in the clear. Observe that $P_{2}$ evaluates the circuit to obtain *y*, masked by common random bit $u_{3}$ of $P_{0}$ and $P_{1}$. In other words, *y* is uniformly random to $P_{2}$. Therefore, the view of A in real execution is computationally indistinguishable from the view of Sim in the ideal execution.    □

**Theorem 5**. *$\Pi_{\mathrm{BitInj}}$ securely realizes the functionality $F_{\mathrm{BitInj}}$ in the $\left(F_{\mathrm{Bit}}2A,F_{\mathrm{Mul}}\right)$-hybrid model and against a semi-honest adversary A, who only corrupts one single party.*

**Proof.**  The security of $\Pi_{\mathrm{BitInj}}$ can be reduced to the security of $\Pi_{\mathrm{Bit}}2A$ and $\Pi_{\mathrm{Mul}}$, which was proven to be secure in Theorem 4 and [19], respectively. Since we make only black-box access to $F_{\mathrm{Bit}}2A$ and $F_{\mathrm{Mul}}$, according to sequential composition, the bit injection protocol we proposed is secure in the semi-honest model.    □

**Theorem 6**. *$\Pi_{\mathrm{Clamp}}$ securely realizes the functionality $F_{\mathrm{Clamp}}$ in the $\left(F_{\mathrm{MSB}},F_{\mathrm{BitInj}}\right)$-hybrid model and against a semi-honest adversary A, who only corrupts one single party.*

**Proof.**  The security of $\Pi_{\mathrm{Clamp}}$ can be reduced to the security of $\Pi_{\mathrm{MSB}}$ and $\Pi_{\mathrm{BitInj}}$, which was proven to be secure in Theorems 4 and 5, respectively. Since we make only black-box access to $F_{\mathrm{MSB}}$ and $F_{\mathrm{BitInj}}$, according to the sequential composition, our secure clamping protocol is secure in the semi-honest model.    □

## 5. Quantized Neural Network Structure

**Figure 3:**
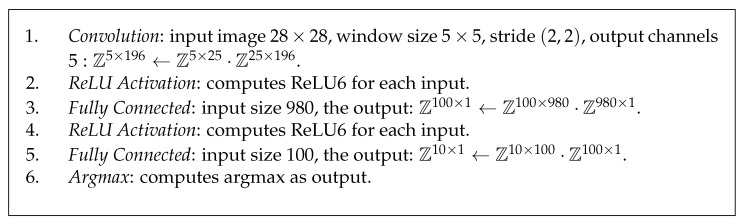
Our QNN structure, where Z denotes the discrete interval ${\left[0,255\right]}_{Z}$.

We consider the convolutional neural network presented in Chameleon [2], which includes a single convolution layer and two fully connected layers. The activation function is ReLU activation. We consider its quantized variant as our QNN structure and describe it in Figure 3. As we pointed out above, we set all data types of QNN from FP32 to INT8.

Instead of evaluating the original ReLU activation, we evaluate ReLU6 activation, as fixed ranges are easier to quantize with high precision in different channels and a quantized model with ReLU6 has less accuracy degradation [22]. Herein, ReLU6 activation is defined as ReLU6(x)=min(max(x,0),6)=Clamp(x;0,6), which is essentially a clamping operation. It seems that we have to invoke a secure comparison protocol to evaluate ReLU6 activation.

In fact, as pointed out by [22], we can take advantage of quantification such that ReLU6 can be entirely fused into the computation of the inner product that precedes it. To do so, we can directly set the quantized parameters to be S=6/255 and Z=0, then α=S(a−Z)∈[0,6] always holds for any $a\in {\left[0,2^{8}\right)}_{Z}$. By doing this, we can clamp the inner product to $a\in {\left[0,2^{8}\right)}_{Z}$, meanwhile evaluating ReLU6 activation. Namely, we can evaluate ReLU6 activation without any communication overhead.

In addition, the evaluation of the argmax function can be computed by invoking the secure comparison protocol.

## 6. Experimental Evaluation

### 6.1. Experimental Setup

We implemented our protocols with Python. All our experiments were executed on a server over Ubuntu 20.04 LTS, which is equipped with Intel(R) Xeon(R) Gold 5222 CPU processor (@3.80GHz) and 32GB RAM memory with AES-NI support. Three parties were simulated by three different terminal ports. We used the Linux traffic tools command tc to simulate LAN and WAN. Specifically, we considered the LAN setting with 625 Mbps bandwidth and 0.2 ms ping time, and the WAN setting with 80 Mbps bandwidth and 20 ms ping time. Note that these parameters are close to the ones we use daily, proving that our solution is practical.

All experiments were executed 10 times on our server to eliminate accidental errors and reported results with the average. We set the bit-length of the shares ℓ=64, the fixed-point precision d=13, and the security parameter κ=128.

To simplify the experiment, we also made the following assumptions:

- We suppose that the input of the user was taken from the MNIST dataset [29], which contains 60,000 training images and 10,000 testing images of handwritten digits. Each image is represented as 28×28 pixel with values between 0 and 255 in greyscale. Note that all greyscales are stored with 8-bit integers already, which eliminates the need for data type conversions.
- We assume that the model owner shared the quantized parameters of each layer among all servers. In short, quantized parameters are encoded to all layers.

### 6.2. Experimental Results for Secure Inference

In our experiment, we compare our solution to two-party framework Chameleon [2] and various three-party frameworks, including Banners [12], SecureBiNN [13] and SecureQ8 [15]. Note that both Chameleon and Banners are not publicly available; hence, we use their reported results directly for reference. Both Banners and SecureBiNN are designed for binary neural network inference. We also compare our solution to SecureQ8, which was also based on INT8 quantized and implemented by MP-SPDZ [30] in the same setting. The experimental results of both LAN and WAN are reported in Table 4. All communication is reported in MB, and runtimes are in seconds.

The author of Chameleon [2] claims that the original network gives us accuracy of 99%. However, our experiment shows that the accuracy is less than 80% when we convert it into a quantized variant as shown in Figure 3. Therefore, instead of reporting the Top-1 accuracy of the model, we reported its Top-5 accuracy, where the truth label is among the first five outputs of the model. In this way, our proposed solution gives us Top-5 accuracy of 98.4%. Note that the reported accuracy of different frameworks is only for reference since it may depend on the model parameters. This is beyond the scope of our work.

As shown in Table 4, almost all quantized frameworks are faster than the nonquantized scheme Chameleon in the same setting. The communication cost of the quantized frameworks is also less than that of the nonquantized scheme. In addition, INT8 quantized schemes are better than binarized schemes in terms of Top-5 accuracy, but the latter have lower communication costs and runtimes.

Compared to Chameleon, due to the quantization technique, our protocols were 1.41 times and 1.94 times faster in the LAN and WAN settings, respectively. In addition, our protocols were 1.32 times lower in online communication. Compared to SecureQ8, our scheme was 1.11 times slower in the LAN setting, but 1.5 times faster in the WAN setting. Because our protocols have constant-round complexity, it is suitable for a low-bandwidth and high-latency network. Note that the online communication costs of our scheme were slightly larger than SecureQ8, as our comparison protocol is based on three-party GC, where the decoding key is related to security parameter κ.

**Figure 4:**
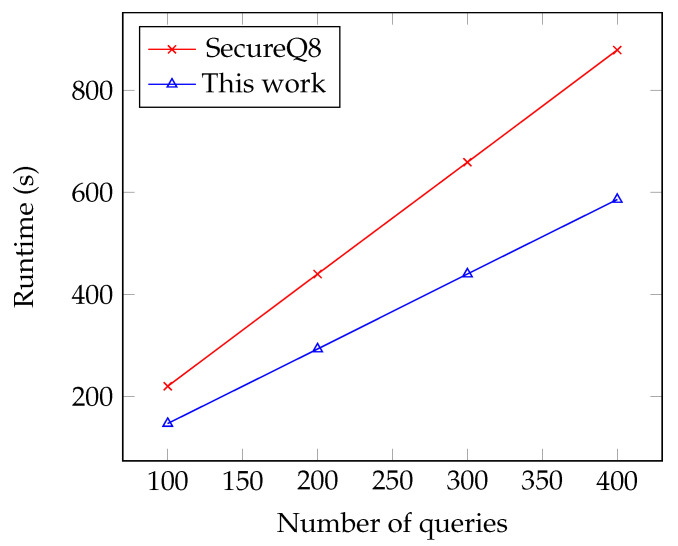
Performance comparison of our solution with SecureQ8 [15] for batch inference over WAN, where query means the input of the user.

Our protocols also enjoy the benefit of the offline–online paradigm. Specifically, most of the communication cost of the online phase is transferred to the offline phase, which makes our scheme more efficient than SecureQ8 in the online phase, especially in the WAN setting. To see this more clearly, we also plot a performance comparison of batch inferences in Figure 4.

## 7. Conclusions

We proposed a series of three-party protocols based on MSS in this study. Our key contribution is more communication-efficient building blocks for QNN inference. Our experiment shows that our protocols are suitable for low-bandwidth and high-latency environments, especially in the WAN network. All these blocks can also be used in other applications as long as the underlying sharing semantics are the same as ours.

Our constant-round comparison protocol is built on GC, and although free of OT, the online communication is related to the security parameter κ. How to construct a constant-round secure comparison protocol such that the online communication cost is independent of security parameters is still an open problem.

Moreover, we only consider a semi-honest adversary with Q3 structures (i.e., the adversary corrupts no more than 1/3 parties). Achieving security against other adversary structures with malicious adversaries will be the future work.

## Figures and Tables

**Table 1:** Description of notations used in this work.

Notation	Description
$\overset{c}{\equiv }$	Computationally indistinguishable
κ	The computational security parameter
$P_{j}$	The computing party, where j∈{0,1,2}
A	The tensor or matrix
a	The vector
*ℓ*	The logarithm of the ring size
$Z_{2^{\ell }},Z_{2}$	The integer ring and the boolean ring
[a,b]	The real interval
${\left[a,b\right]}_{Z}$	The discrete interval [a,b]∩Z
$x\in_{R}D$	Uniform random sample *x* from distribution *D*
$\left(a\leq_{?}b\right)$	Return 1 if a≤b holds, and 0 otherwise
Clamp(x;a,b)	Set x←a if x<a, x←b if x>b, and x←x otherwise

**Table 2:** The shares of different secret-sharing schemes for each party, where *x* is the secret.

Scheme	Notation	$P_{0}$	$P_{1}$	$P_{2}$
ASS	$\langle x\rangle :=({\langle x\rangle }_{1},{\langle x\rangle }_{2})$	—	$x_{1}$	$x_{2}$
MSS	$〚x〛:=(m_{x},{\langle \lambda_{x}\rangle }_{1},{\langle \lambda_{x}\rangle }_{2})$	$\left({\langle \lambda_{x}\rangle }_{1},{\langle \lambda_{x}\rangle }_{2}\right)$	$\left(m_{x},{\langle \lambda_{x}\rangle }_{1}\right)$	$\left(m_{x},{\langle \lambda_{x}\rangle }_{2}\right)$

**Table 3:** The communication and round complexity of our protocols, where *ℓ* denotes the logarithm of the ring size, and κ denotes security parameter. All communications are reported in a number of bits.

Protocol	Offline		Online	
	Communication	Rounds	Communication	Rounds
$\Pi_{\mathrm{Mul}}$	*ℓ*	1	2ℓ	1
$\Pi_{\mathrm{Share}}$	0	0	2ℓ	1
$\Pi_{\mathrm{Trunc}}$	*ℓ*	1	2ℓ	1
$\Pi_{\mathrm{Bit}}2A$	2ℓ	1	2ℓ	1
$\Pi_{\mathrm{MSB}}$	5κℓ	1	κℓ+2	2
$\Pi_{\mathrm{BitInj}}$	—	—	4ℓ	2
$\Pi_{\mathrm{Clamp}}$	—	—	2κℓ+12ℓ+4	8

**Table 4:** Performance comparison of our solution with other frameworks for classifying a single image from the MNIST dataset, where Top-5 accuracy means the truth label is among the first 5 outputs of the model. (*): Banners and SecureQ8 were only reported in the online phase. (**): No offline phase is required in SecureBiNN.

Framework	Quantized	Secret Sharing	Top-5 Accuracy	Runtime (s)				Communication (MB)	
				LAN		WAN			
				Offline	Online	Offline	Online	Offline	Online
Chameleon [2]	FP32	ASS	99.0%	1.254	0.991	4.028	2.851	7.798	5.102
Banners * [12]	Binary	RSS	97.3%	—	0.120	—	—	—	2.540
SecureBiNN ** [13]	Binary	RSS	97.2%	—	0.007	—	0.440	—	0.032
SecureQ8 * [15]	INT8	RSS	98.4%	—	0.629	—	2.198	—	3.523
This work	INT8	MSS	98.4%	1.018	0.701	3.279	1.465	5.982	3.853

## Data Availability

Not applicable.

## Abbreviations

The following abbreviations are used in this manuscript:

MLaaS	Machine Learning as a Service
QNN	Quantized Neural Network
FHE	Fully Homomorphic Encryption
MPC	Secure Multiparty Computation
SS	Secret Sharing
ASS	Additive Secret Sharing
RSS	Replicated Secret Sharing
MSS	Masked Secret Sharing
GC	Garbled Circuit
OT	Oblivious Transfer
PRF	Pseudo-Random Function
LAN	Local Area Network
WAN	Wide Area Network
FP32	32-bit Floating-Point
INT8	8-bit Integer
ReLU	Rectified Linear Unit
MSB	Most Significant Bit
PPA	Parallel Prefix Adder

## Appendix A. Correlated Randomness

In some protocols, each pair of parties needs to generate the same randomness. To reduce communication costs and rounds of the protocol, similar to the prior works [7,8,19,20,25], we define functionality $F_{\mathrm{Rand}}$, which allows the parties to generate the same randomness.

Let $F:{\left\{0,1\right\}}^{\kappa }\times {\left\{0,1\right\}}^{\kappa }\to R$ be a secure PRF known to all parties, where R is the computation field $Z_{2^{\ell }}$ or $Z_{2}$ in this study. Suppose that each pair of parties $\left(P_{i},P_{j}\right)$ holds a preshared random PRF key $k_{ij}\in_{R}{\left\{0,1\right\}}^{\kappa }$ and maintains a local counter ctr as the number of PRF invoking, where $k_{ij}$ can be generated by using the Diffie–Hellman key exchange protocol between $P_{i}$ and $P_{j}$ in a one-time setup, and PRF can be implemented by an advanced encryption standard in counter mode [31].

To achieve $F_{\mathrm{Rand}}$, $\Pi_{\mathrm{Rand}}$ can be performed by letting $P_{i}$ and $P_{j}$ locally compute $r\leftarrow F(k_{ij},\mathrm{ctr})$.

## References

[1] Ribeiro M., Grolinger K., Capretz M.A. MLaaS: Machine Learning as a Service. Proceedings of the 2015 IEEE 14th International Conference on Machine Learning and Applications (ICMLA).

[2] Riazi M.S., Weinert C., Tkachenko O., Songhori E.M., Schneider T., Koushanfar F. Chameleon: A Hybrid Secure Computation Framework for Machine Learning Applications. Proceedings of the 2018 on Asia Conference on Computer and Communications Security—ASIACCS ’18.

[3] Huang Z., Lu W.J., Hong C., Ding J. Cheetah: Lean and fast secure Two-Party deep neural network inference. Proceedings of the 31st USENIX Security Symposium (USENIX Security 22).

[4] Wang Y., Luo Y., Liu L., Fu S. pCOVID: A Privacy-Preserving COVID-19 Inference Framework. Proceedings of the Algorithms and Architectures for Parallel Processing.

[5] European Union (2016). General Data Protection Regulation (GDPR). https://gdpr-info.eu/.

[6] Gilad-Bachrach R., Dowlin N., Laine K., Lauter K., Naehrig M., Wernsing J. CryptoNets: Applying Neural Networks to Encrypted Data with High Throughput and Accuracy. Proceedings of the 33rd International Conference on Machine Learning.

[7] Mohassel P., Rindal P. ABY3: A Mixed Protocol Framework for Machine Learning. Proceedings of the 2018 ACM SIGSAC Conference on Computer and Communications Security.

[8] Wagh S., Tople S., Benhamouda F., Kushilevitz E., Mittal P., Rabin T. (2021). Falcon: Honest-Majority Maliciously Secure Framework for Private Deep Learning. Proc. Priv. Enhancing Technol..

[9] Rouhani B.D., Riazi M.S., Koushanfar F. Deepsecure: Scalable provably-secure deep learning. Proceedings of the 55th Annual Design Automation Conference.

[10] Mohassel P., Zhang Y. SecureML: A system for scalable privacy-preserving machine learning. Proceedings of the 2017 IEEE Symposium on Security and Privacy (SP).

[11] Riazi M.S., Samragh M., Chen H., Laine K., Lauter K.E., Koushanfar F. XONN: XNOR-based oblivious deep neural network inference. Proceedings of the 28th USENIX Security Symposium, USENIX Security 2019.

[12] Ibarrondo A., Chabanne H., Önen M. Banners: Binarized Neural Networks with Replicated Secret Sharing. Proceedings of the 2021 ACM Workshop on Information Hiding and Multimedia Security.

[13] Zhu W., Wei M., Li X., Li Q. SecureBiNN: 3-Party Secure Computation for Binarized Neural Network Inference. Proceedings of the Computer Security—ESORICS 2022.

[14] Agrawal N., Shahin Shamsabadi A., Kusner M.J., Gascón A. QUOTIENT: Two-Party Secure Neural Network Training and Prediction. Proceedings of the 2019 ACM SIGSAC Conference on Computer and Communications Security.

[15] Dalskov A., Escudero D., Keller M. (2020). Secure Evaluation of Quantized Neural Networks. Proc. Priv. Enhancing Technol..

[16] Shen L., Dong Y., Fang B., Shi J., Wang X., Pan S., Shi R. ABNN2: Secure two-party arbitrary-bitwidth quantized neural network predictions. Proceedings of the 59th ACM/IEEE Design Automation Conference.

[17] Keller M., Sun K. Secure Quantized Training for Deep Learning. Proceedings of the 39th International Conference on Machine Learning.

[18] Goldreich O. (2004). The Foundations of Cryptography—Volume 2: Basic Applications.

[19] Chaudhari H., Choudhury A., Patra A., Suresh A. ASTRA: High Throughput 3PC over Rings with Application to Secure Prediction. Proceedings of the 2019 ACM SIGSAC Conference on Cloud Computing Security Workshop.

[20] Wagh S., Gupta D., Chandran N. (2019). SecureNN: 3-Party Secure Computation for Neural Network Training. Proc. Priv. Enhancing Technol..

[21] Guo Y. (2018). A Survey on Methods and Theories of Quantized Neural Networks. arXiv.

[22] Jacob B., Kligys S., Chen B., Zhu M., Tang M., Howard A., Adam H., Kalenichenko D. Quantization and Training of Neural Networks for Efficient Integer-Arithmetic-Only Inference. Proceedings of the 2018 IEEE/CVF Conference on Computer Vision and Pattern Recognition.

[23] Ádám Mann Z., Weinert C., Chabal D., Bos J.W. (2022). Towards Practical Secure Neural Network Inference: The Journey So Far and the Road Ahead, Cryptology ePrint Archive, Paper 2022/1483. https://eprint.iacr.org/2022/1483.

[24] Ohata S., Nuida K. Communication-Efficient (Client-Aided) Secure Two-Party Protocols and Its Application. Proceedings of the Financial Cryptography and Data Security.

[25] Patra A., Suresh A. BLAZE: Blazing Fast Privacy-Preserving Machine Learning. Proceedings of the 2020 Network and Distributed System Security Symposium.

[26] Kolesnikov V., Schneider T. Improved Garbled Circuit: Free XOR Gates and Applications. Proceedings of the Automata, Languages and Programming.

[27] Zahur S., Rosulek M., Evans D. Two Halves Make a Whole. Proceedings of the Advances in Cryptology—EUROCRYPT 2015.

[28] Canetti R. Universally Composable Security: A New Paradigm for Cryptographic Protocols. Proceedings of the 42nd IEEE Symposium on Foundations of Computer Science.

[29] Yann L., Corinna C., Chris B. (2017). The MNIST Dataset of Handwritten Digits. http://yann.lecun.com/exdb/mnist/.

[30] Keller M. MP-SPDZ: A Versatile Framework for Multi-Party Computation. Proceedings of the 2020 ACM SIGSAC Conference on Computer and Communications Security.

[31] Katz J., Lindell Y. (2020). Introduction to Modern Cryptography.

